# Supplementary material for: Perceptions of barriers and facilitators to opioid reduction after total joint arthroplasty among orthopedic surgeons practicing in Canada, Japan, and the Netherlands: A qualitative description study
Source: PLoS One. 2025 Aug 29;20(8):e0331335. doi: 10.1371/journal.pone.0331335 (PMC12396690; doi:10.1371/journal.pone.0331335)
Supplement: S1 File — (DOCX) [file pone.0331335.s001.docx]

**S1 File - Semi-Structured Interview Guide (30-45 Minutes)**

**Participant ID: Date:**

**Location/Country: Researcher:**

**Start time: End time:**

**Date of informed consent:**

*Do you have any questions about the information letter that was sent earlier?*

*Please note: (i) we will remove any identifying information from the transcripts and will use your interview responses for research purposes only, and (ii) you can skip any questions that you do not wish to answer or stop the interview at any time. (iii) We may publish direct quotes from your interview, but we will not publish any information that can identify you personally. Do you agree to be interviewed?* **(Yes) or (No)**

OK to audio record (circle): **Y / N**

OK to video record [*if on Zoom*] (circle): **Y / N**

*I will be asking you some in-depth and probing questions about your perceptions and experiences about opioid prescribing after total joint replacement. Please answer as honestly as you can, and you may prefer to skip some questions. We are using the data we collect for research purposes only. Also, please feel free to ask me if you want any clarifications at any time. Before we begin, do you have any questions?*

**Semi-structured interview questions:**

Demographics

1. What is your gender?
2. For how long have you been practicing as an orthopedic surgeon (in years)?
3. Do you work in an academic or community setting?
4. Approximately how many total knee arthroplasty procedures do you do in a year?
5. Approximately how many total hip arthroplasty procedures do you do in a year?
6. On average, approximately what percent of your total joint arthroplasty patients get opioid prescriptions from you or your team?
7. What is the typical length of stay for a patient after total hip arthroplasty in your setting?
   1. What is the typical length of stay for a patient having total knee arthroplasty?

Pain questions (yellow - do not necessarily have to ask if they do not prescribe opioids at all)

1. Please describe your practices for pain management (out of hospital or after discharge) for total joint arthroplasty patients.
   1. *If no mention of non-pharmacological alternatives* - Do you use non-pharmacological alternatives for pain management? If so, please describe. (i.e. ice, compression, physical therapy etc.)
   2. Do you have any patients with substance use disorder?
      1. *If yes* - Do you change your practice of pain management for these patients? If so, how?
2. Do you do direct anterior hip replacement surgeries?
   1. *If yes* – Do you change your pain management method for people with direct anterior hips compared to those with other approaches?
   2. If so, how?
3. Have you made any changes to your opioid prescribing practices over the years? If so, how? and why?
4. What challenges are orthopedic surgeons facing right now in terms of prescribing opioids and managing post-operative pain, if any?
5. Do you or any of your team members (e.g. nurse, physician assistant, etc.) provide patients with education about post-operative pain management?
   1. *If yes* - Please describe the education you provide.
      1. When do you usually provide this education to patients (i.e. before or after surgery)?
   2. *If no -* Do you think that TJA patients should be informed or educated about their pain management? If so, when and how?
6. What is the typical length or quantity and dose of opioids that you give to patients?
7. How do you decide the length of opioid prescriptions that you give to patients?
   1. *If they have a standard prescription* – how did you determine or decide on this standard prescription length?
   2. Do you provide instructions to patients on how to manage unused opioid medications? If so, please describe.
8. What do you do if the patient you prescribed opioids to still has pain after the prescription is done? (*probe* - do you change or extend the prescription?)
   1. What percent of patients request refills for prescribed opioid medications?
   2. When patients request a refill of opioids, how do you decide to refill or not?
9. Have you received any continuing education related to opioid prescribing?
   1. *If yes* - what continuing education have you received (i.e. seminars, courses, etc.)?
   2. *If no* - Do you think that continuing education related to opioid prescribing would be helpful? Why?
      1. *If yes* - Describe the education that would be most useful for you.
10. Do you think that there are differences between you and other orthopedic surgeons in opioid prescribing for joint arthroplasty at your hospital?
    1. *If yes* - why do you think some surgeons in your hospital prescribe more or fewer opioids than others?
    2. Are there any policies and guidelines that you use to support or guide you in prescribing opioids?
11. In general, do you think that opioids are appropriately prescribed for joint arthroplasty patients by surgeons in your hospital/institution?
    1. *If no* - Why?
12. Do you have any additional thoughts or comments about opioid prescribing for total joint arthroplasty procedures that we haven’t yet discussed? Anything else that came to mind during the interview?

We are looking for more surgeons to interview. Could you suggest other orthopedic surgeons that you know who would be good to speak with about the use of opioids after total joint arthroplasty? If so, can you provide contact information for this/these individual(s)?

Is it okay if we refer to you by name when we contact these individuals? ***Y/N***

*Would you agree to review, and confirm, our findings from your interview?* ***Y/N***

*Thank you for your time.*
