# Supplementary material for: Perceptions of barriers and facilitators to opioid reduction after total joint arthroplasty among orthopedic surgeons practicing in Canada, Japan, and the Netherlands: A qualitative description study
Source: PLoS One. 2025 Aug 29;20(8):e0331335. doi: 10.1371/journal.pone.0331335 (PMC12396690; doi:10.1371/journal.pone.0331335)
Supplement: S2 File — (DOCX) [file pone.0331335.s002.docx]

**S2 File – Interviewer Positionality Statements**

**Interviewer 1:**

I identify as a South-Asian, Canadian, graduate student, and am able-bodied and cis-gendered (female). Considering my experiences and views, I find constructivism relevant as I believe that all truth is relative and socially constructed by individuals (i.e. subjective experiences of pain). Additionally, in light of my background in quantitative research, and consistent with Thorne (2016), I believe that the findings of a study should be treated as though they will be applied in practice, and should not merely be theoretical (pragmatism). Accordingly, my choice of research design, as guided by my beliefs and values, would be an interpretive description approach. This is primarily because the idiosyncratic experiences of pain constitute multiple constructed realities. Furthermore, as a result of my experiences, I believe that reality is complex, contextual, constructed, and ultimately subjective. As I will be using an interpretive description approach, semi-structured, 1:1 interviews are the most optimal data sources.

Presently, I am a graduate student in the Health Research Methodology program, and am not a health care practitioner and do not have a professional background. Accordingly, while carrying out the data collection phase of the study, I perceive that the participants will provide me with broader, non-health-related content during the interviews as I do not have a professional role, and some participants may have inherent biases relating to my role as a student. Moreover, as I do not have a professional identity or title, my gender (female) and relatively young age (22 years) would be key variables that would interact with and influence the interview process. For example, it is plausible that communication during the interview may be more colloquial than it would be if the interviewer were a practicing professional, regarded as being at a higher social and professional status than me. Additionally, as I would be in a position of an “outsider” in terms of not being a practicing medical professional and not having first-hand experience with opioids, there will be some limitations in my ability to fully understand the formal and informal aspects of opioid prescribing and certain situations. Subsequently, there may be a barrier to recognizing more subtle language use, associations, jargon, and cues. Although having second-hand experience with post-operative pain and opioids (due to my extensive observations of my grandparents who were reliant on opioids for their post-TKA pain) is not equivalent to being afflicted with pain or using opioids directly, I can approach the process of data collection and analysis with some knowledge about this subject. Likewise, I have done several projects pertaining to the opioid crisis and assessing the efficacy of specific non-opioid alternatives for pain. However, the risk of “blurring boundaries” between the interviewer and interviewee would be minimized as I would be coming from an “outsider” perspective. In spite of this, being partly “ignorant” of the phenomenon puts the respondent in an expert position, and this may, in turn, allow me to approach data collection and analysis from a novel viewpoint.

In light of my extensive background in pain research and related personal experiences, I may be biased and inclined to focus more on the positive perceptions of surgeons who recognize that there is an opioid crisis and their practice considers its implications as they heed to all guidelines (potentially due to the social desirability bias). I believe that opioids are associated with numerous adverse events and surgeons should avoid using opioids when possible. Therefore, a constant deliberate effort will be necessitated to ensure that I am precisely documenting the complete narrative, and I must scrutinize how I filter the interviewee’s responses through the lens of my experiences.

**Interviewer 2:**

I identify as a cis-gendered male, able-bodied Canadian undergraduate student. My experiences and views align with the constructivism paradigm as I believe that truth is relative to the individual. In terms of the study's results, I hold a pragmatistic view that the study's findings should be treated as though they will be applied in practice.

As an undergraduate student in the Health Science program, I am not a health care practitioner and do not have a professional background. Consequently, I presume that the participants may provide me with simplified content during the interviews as I do not have a professional role and am relatively young (19 years old) compared to the participants. Due to my lack of professional background in the medical profession, I may have some limitations in my ability to understand certain aspects of opioid prescribing. I may also have difficulty understanding subtle language use, associations, jargon, and cues.

I do not have any personal experience or formal education on the topic of Opioids. However, through past article readings, I have come across Canada's Opioid Crisis and the devastating effects opioids have on individuals, families, and communities across Canada. Therefore, I may be inclined to pay focus on surgeons who recognize that there is an opioid crisis. On the other hand, I recognize that I do not know the full extent of the benefits that Opioids offer in particular medical applications. From my perspective, while Opioids may offer many benefits, medical professionals should limit their opioid usage unless deemed necessary. Ultimately, I will need to inspect how I filter the interviewee's responses through the lens of my experiences and ensure that I am precisely documenting the complete narrative.
