# Supplementary material for: Perceptions of barriers and facilitators to opioid reduction after total joint arthroplasty among orthopedic surgeons practicing in Canada, Japan, and the Netherlands: A qualitative description study
Source: PLoS One. 2025 Aug 29;20(8):e0331335. doi: 10.1371/journal.pone.0331335 (PMC12396690; doi:10.1371/journal.pone.0331335)
Supplement: S3 File — S3 Table 1. Pain medications prescribed by orthopaedic surgeons in Canada, Japan, and the Netherlands. S3 Table 2. Facilitators and barriers to reduced opioid prescribing influencing surgeons’ prescribing practises. S3 Table 3. Facilitators and barriers to reduced opioid prescribing related to patient factors/perspectives. S3 Table 4. Facilitators and barriers to reduced opioid prescribing related to providing collaborative patient care. S3 Table 5. Facilitators and barriers to reduced opioid prescribing related to policies, guidelines, and regulations on opioid prescribing. S3 Table 6. Barriers and facilitators to reduced opioid prescribing related to opioid risk mitigation via surgeon education. S3 Table 7. Surgeons’ personal perceptions and beliefs on opioid prescribing. (DOCX) [file pone.0331335.s003.docx]

**S3 File**

**Table 1** Pain medications prescribed by orthopaedic surgeons in Canada, Japan, and the Netherlands

| **Country** | **Pain Medications Prescribed** |
| --- | --- |
| **Canada** | Acetaminophen  NSAIDs  Celecoxib  Morphine  Oxycodone/paracetamol  Oxycodone  Hydromorphone  Tramadol  Pregabalin  Gabapentin |
| **Japan** | NSAIDs (e.g. Oxaprozin, Ibuprofen, Diclofenac, etc.)  Celecoxib  Acetaminophen  Tramadol  Serotonin and norepinephrine reuptake inhibitors (SNRIs)  Fentanyl  Tramocet |
| **Netherlands** | NSAIDs (e.g. Diclofenac, Naproxen, etc.)  Paracetamol  Celecoxib  Oxycodone  Morphine  Tramadol |

*NSAIDs = Nonsteroidal anti-inflammatory drugs*

**Table 2** Facilitators and barriers to reduced opioid prescribing influencing surgeons’ prescribing practises

|  | **Canada** | **Japan** | **The Netherlands** |
| --- | --- | --- | --- |
| **Facilitators** | - Decreased opioids quantity and length over years of practise - Considers patients’ individual needs, characteristics, and medical history when prescribing or refilling - Use of multimodal strategies for pain control - Non-opioid analgesia and non-pharmacological strategies are always used - PRN (use as needed) - Wary of the side-effects of opioids (particularly addiction and misuse) | - Belief that opioids are unnecessary for managing post-operative pain (i.e. rarely prescribed post-operatively and post-discharge) - Heavy reliance on NSAIDs or acetaminophen - Ladder for pain management (non-opioid analgesia first, opioids as a last resort) - Non-pharmacological strategies are often used - Considers patients’ individual needs, characteristics, and medical history when prescribing or refilling - Regular monitoring of patients’ pain levels - Use of multimodal strategies for pain control - Wary of the side-effects of opioids (addiction, falls, sedation, nausea, constipation, hepatic dysfunction) | - Decreased opioids quantity and length over years of practise - Standard institutional post-operative/discharge pain management is followed and revised regularly - Standard perioperative protocol decreases post-discharge opioid use - Considers patients’ individual needs, characteristics, and medical history when prescribing or refilling - Use of multimodal strategies for pain control - Non-pharmacological strategies are often used - Ladder for pain management (non-opioid analgesia first, opioids as a last resort) - PRN (use as needed) - Wary of the side-effects of opioids (particularly addiction and misuse) - Almost no perceived variation in opioid prescribing among in-hospital colleagues - Pharmacy instructs patients to return unused opioids |
| **Barriers** | - Concerns with side-effects of non-opioid analgesics (i.e. NSAIDs, acetaminophen) - Balancing patients’ needs for pain relief and mobility, and risks of opioids (tension from competing pressures) - Lack of effective alternative strategies for managing post-operative pain - Lack of institutional standard protocols for opioid prescribing - Busy schedule and lack of time to monitor patients’ use or for long consultations - Outdated prescribing practises and resistance to opioid reduction among senior orthopaedic surgeons - Differences in individual surgeons’ preferences, experiences, and training may drive variation in prescribing - No instructions given to patients on what to do with unused opioids | - Concerns with side-effects of non-opioid analgesics (i.e. NSAIDs, acetaminophen) - Use of opioids pre-operatively due to long wait times for surgery - Lack of an institutional standard protocol for managing pain post-operatively and prescribing opioids | - Concerns with side-effects of non-opioid analgesics (i.e. NSAIDs, paracetamol) - Balancing patients’ needs for pain relief and mobility, and risks of opioids (tension from competing pressures) - Busy schedule and lack of time to monitor patients’ use or for long consultations - Unfamiliar with the specific details of the institutional standard pain management protocol, as the anesthesiologist is largely responsible for immediate post-op prescribing |

**Table 3** Facilitators and barriers to reduced opioid prescribing related to patient factors/perspectives

|  | **Canada** | **Japan** | **The Netherlands** |
| --- | --- | --- | --- |
| **Facilitators** | - Provides pre-operative and post-operative education to patients about their pain expectations, side-effects, medications | - Cultural resistance to opioid use among Japanese patients (preference for non-opioid pain management options) - Patient satisfaction with pain management using weak opioids or non-opioid analgesia - No unused opioids leftover | - Provides pre-operative and post-operative education to patients about their pain expectations, side-effects, medications, pain management ladder, opioid tapering |
| **Barriers** | - Concerns with patients’:   - Dissatisfaction with less quantity/dosing of opioids   - Preference for opioids rather than alternatives   - Expectation of being pain-free after surgery   - Tolerance to pain   - Access to non-pharmacological or over-the-counter prescriptions (financial barriers) - Patients may not return unused opioids and divert them for misuse | - Many patients do not receive education about their post-operative pain - Perception that patient education about pain management is unnecessary or only required when prescribing strong opioids | - Patients may not return unused opioids and divert them for misuse - Concerns with patients’:   - Dissatisfaction with less quantity/dosing of opioids   - Preference for opioids rather than alternatives   - Expectation of being pain-free after surgery   - Perceptions of pain - Patients concerned with side-effects of non-opioid analgesics (i.e. NSAIDs) |

**Table 4** Facilitators and barriers to reduced opioid prescribing related to providing collaborative patient care

|  | **Canada** | **Japan** | **The Netherlands** |
| --- | --- | --- | --- |
| **Facilitators** | - Collaboration with other healthcare providers, pharmacy, and pain clinics on:   - Patient education   - Surgeon education   - Patients’ pain management   - Caring for SUD patients   - Development of a standard pain management protocol | - Collaboration with anesthesiologists in decisions regarding patients’ prescriptions, and surgeon and patient education | - Collaboration with other healthcare providers, pharmacy, and pain clinics on:   - Patient education   - Surgeon education   - Patients’ pain management   - Caring for SUD patients   - Development of a standard pain management protocol |
| **Barriers** | - Long wait times for pain clinic - Invisible sources of opioids (concerns with patients receiving refills from other HCPs) | - Lack of a specialized pain management team/clinics - Invisible sources of opioids (concerns with patients receiving refills from other HCPs) | - Invisible sources of opioids (concerns with patients receiving refills from other HCPs) - Variability in the prescribing patterns of other HCPs on the care team |

SUD: substance use disorder

**Table 5** Facilitators and barriers to reduced opioid prescribing related to policies, guidelines, and regulations on opioid prescribing

|  | **Canada** | **Japan** | **The Netherlands** |
| --- | --- | --- | --- |
| **Facilitators** | - Provincial campaigns to decrease opioid prescribing - Institutional regulations and monitoring - Reluctance to prescribe opioids due to fear of litigation or licence suspension | - Anesthesiologists have guidelines on opioid use - Existence of chronic pain guidelines and Japanese Orthopaedic Association Guidelines - Strict regulations on using strong opioids (e.g. morphine, oxycodone, etc.) - Hospital’s pharmaceutical department monitors opioid prescriptions - Some strong opioids are not covered by Japanese health insurance systems for non-cancer pain (e.g. Oxycodone) | - Hospital’s pharmaceutical department monitors opioid prescriptions - Institutional regulations and monitoring |
| **Barriers** | - Outdated policies and guidelines - Many surgeons unaware of policies and guidelines for opioid prescribing - Pressure on surgeons to adhere to strict opioid prescription policies may hinder individualized care (e.g. for patients with severe levels of pain) | - Many surgeons unaware of policies and guidelines for opioid prescribing | - Many surgeons unaware of policies and guidelines for opioid prescribing |

**Table 6** Barriers and facilitators to reduced opioid prescribing related to opioid risk mitigation via surgeon education

|  | **Canada** | **Japan** | **The Netherlands** |
| --- | --- | --- | --- |
| **Facilitators** | - Involvement in opioid-related research and/or groups/societies - Institutional/departmental training on pain management and opioid prescribing (modules, meetings, rounds, conferences) | - E-learning modules must be completed for each specific opioid surgeon prescribes - Pharmaceutical company provides education to surgeons (e.g. via brochures) - Institutional/departmental training and seminars | - Involvement in opioid-related research and/or groups/societies - Institutional/departmental training on pain management and opioid prescribing (modules, meetings, conferences) |
| **Barriers** | - Insufficient surgeon training on strategies for identifying abuse, managing pain for vulnerable populations, dosing, non-opioid alternatives, and trends - Busy schedules of surgeons may decrease attendance to traditional, in-person educational sessions - Some surgeons have not received any continuing education/training on opioid prescribing, and/or perceive it as unnecessary | - Many surgeons have not received any formal continuing education on prescribing opioids - Insufficient education on SUD (e.g. risk factors, management, etc.) | - Insufficient surgeon training on multimodal strategies, updates, novel products, non-opioid alternatives - Some surgeons have not received any continuing education/training on opioid prescribing, and/or perceive it as unnecessary |

SUD: substance use disorder

**Table 7** Surgeons’ personal perceptions and beliefs on opioid prescribing

|  | **Canada** | **Japan** | **The Netherlands** |
| --- | --- | --- | --- |
| **Perceptions/beliefs on opioid prescribing** | - Opioids are not effective in managing post-operative pain - Differences in the addiction potentials of opioids - Not possible to completely eliminate opioids in managing post-arthroplasty pain - Feeling of regret for over-prescribing - TKA is more painful than THA and requires more opioids - Opioids are necessary for improving patients’ sleep and mobility | - Tramadol is not as addictive as other opioids - TKA is more painful than THA - Knowledge gaps and uncertainty surrounding SUD - Need for stronger and longer durations of opioids - Direct anterior approach is less painful than posterior approach - North American and European surgeons encounter more SUD concerns | - Differences in post-op pain levels between private/community and academic hospitals - Differences in the addiction potentials of opioids - Knowledge gaps and uncertainty surrounding SUD - America has more serious problems with the opioid crisis - Not possible to completely eliminate opioids in managing post-arthroplasty pain - TKA is more painful than THA and requires more opioids - Opioids are necessary for improving patients’ sleep and mobility |

SUD: substance use disorder


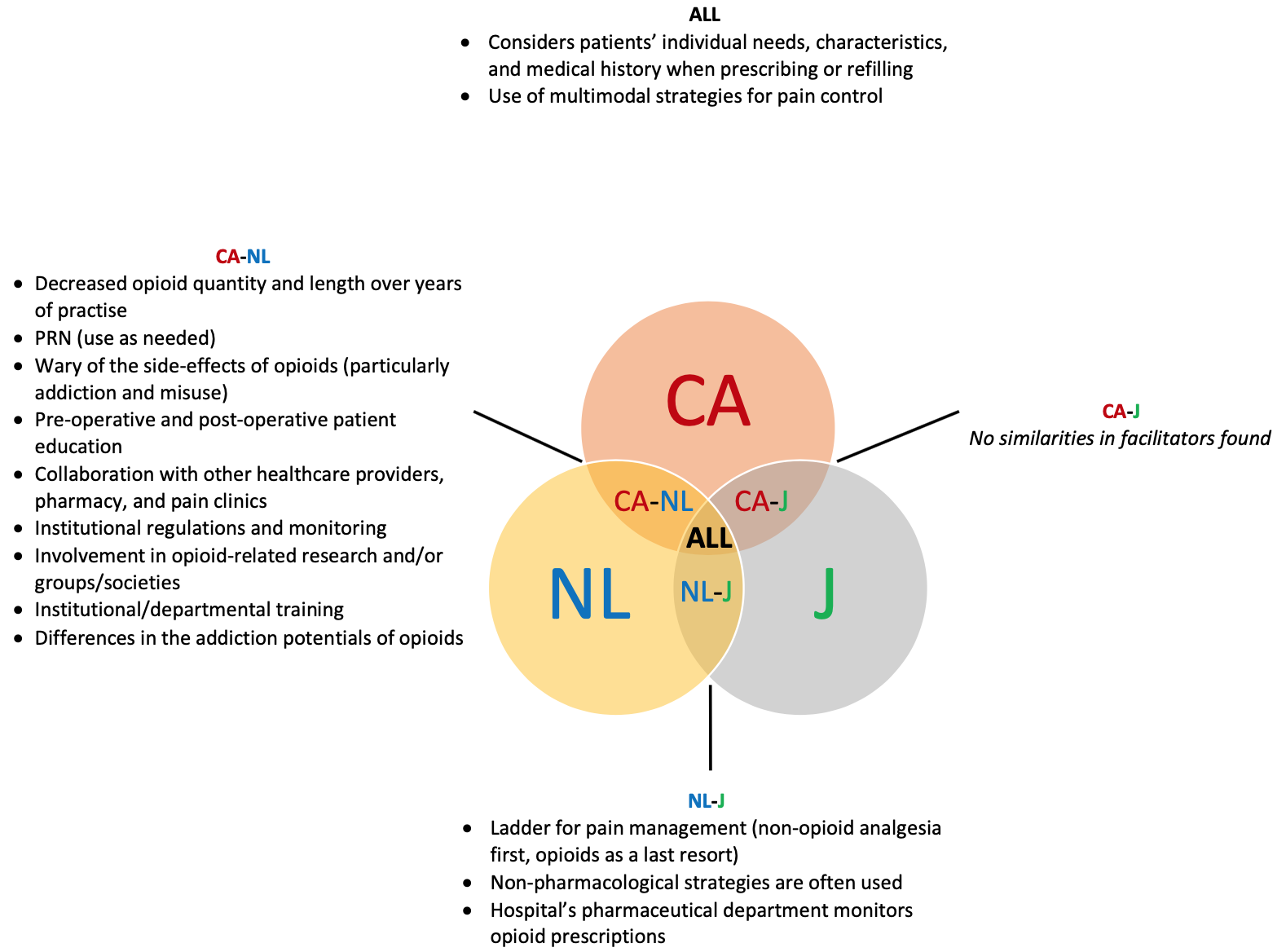


**Fig 1** Comparison of factors perceived by surgeons as facilitating opioid reduction in Canada, Japan, and the Netherlands


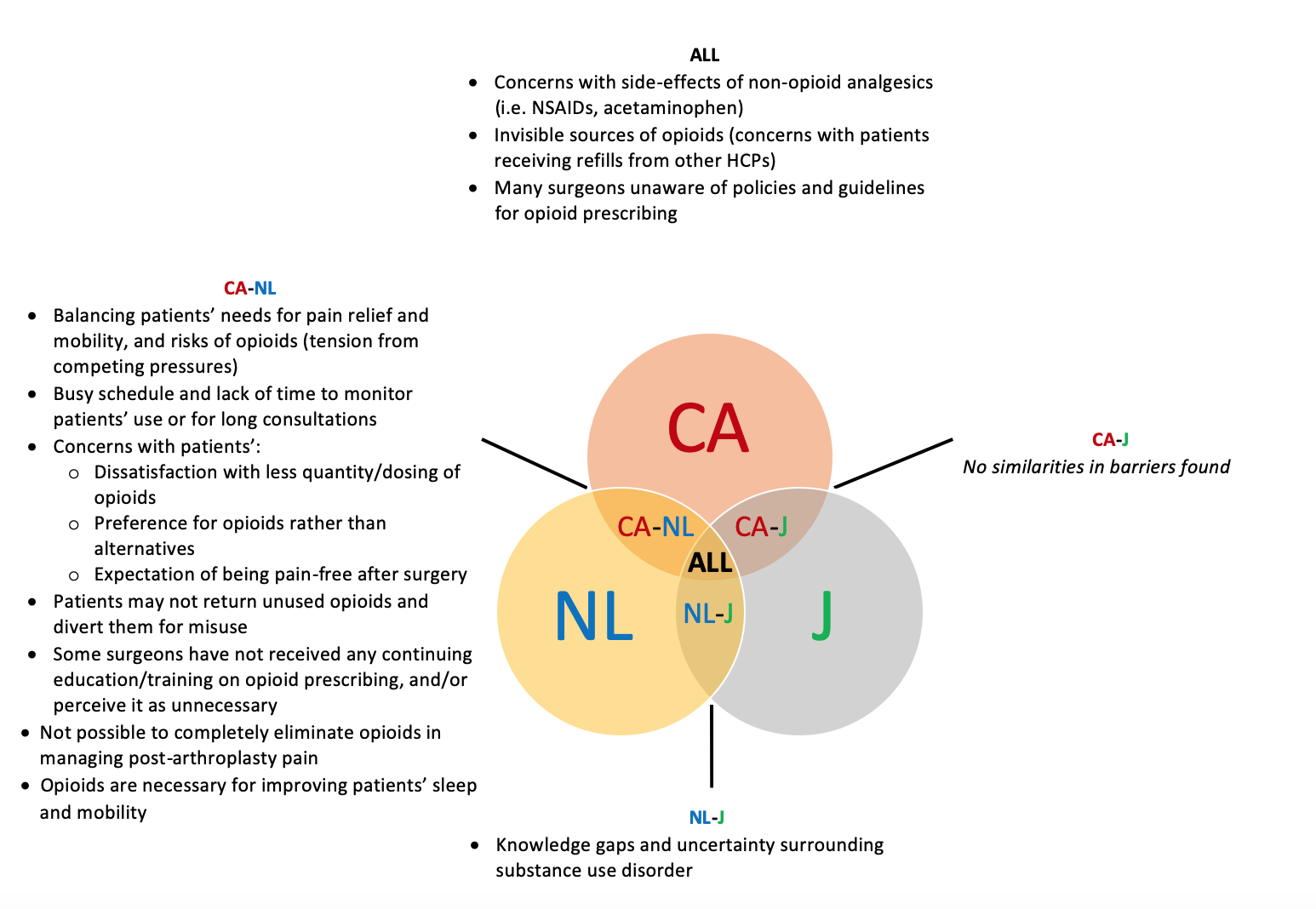


**Fig 2** Comparison of factors perceived by surgeons as hindering opioid reduction in Canada, Japan, and the Netherlands
