# Supplementary figures and images for: Perceptions of barriers and facilitators to opioid reduction after total joint arthroplasty among orthopedic surgeons practicing in Canada, Japan, and the Netherlands: A qualitative description study
Source: PLoS One. 2025 Aug 29;20(8):e0331335. doi: 10.1371/journal.pone.0331335 (PMC12396690; doi:10.1371/journal.pone.0331335)

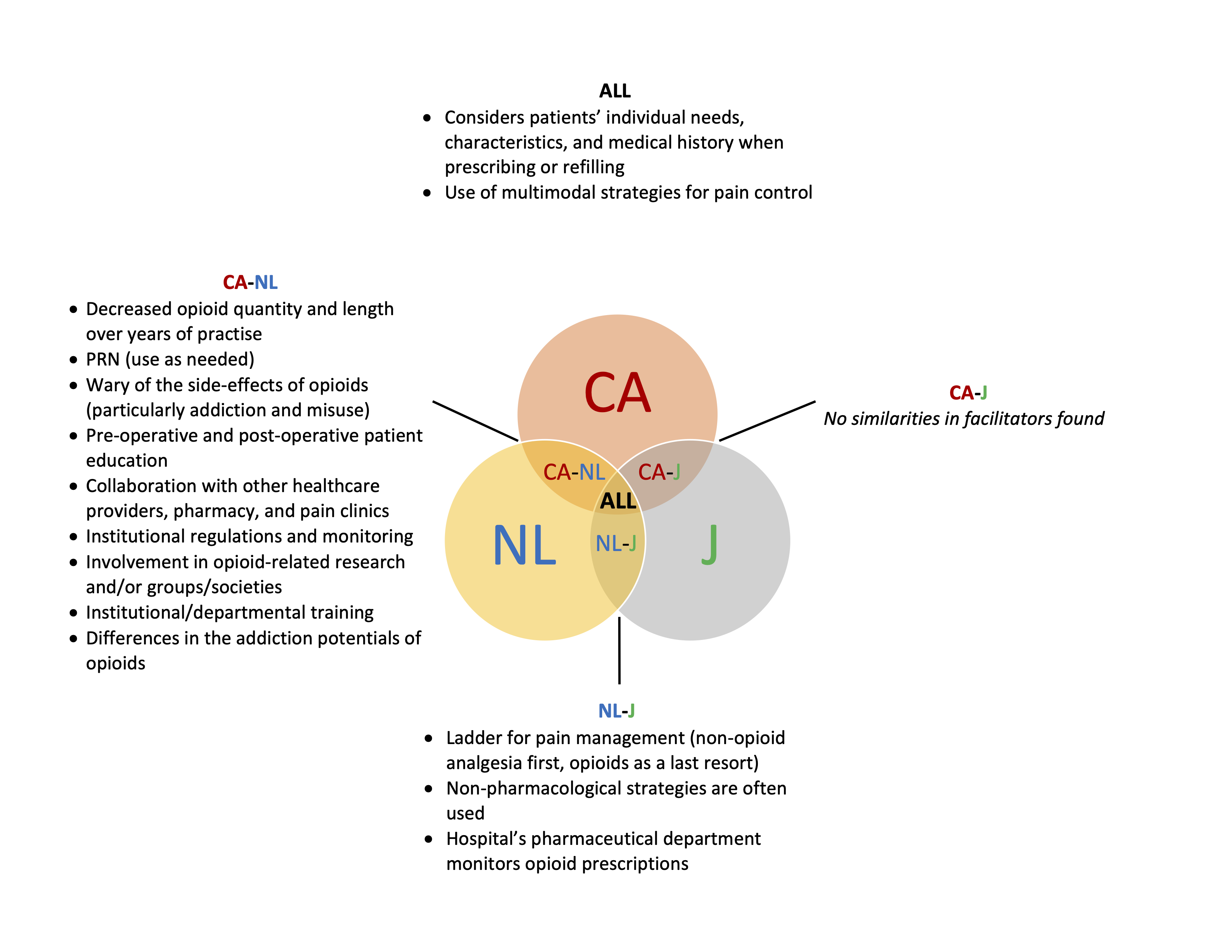

Supplement: S1 Fig — (TIFF) [file pone.0331335.s004.tiff]

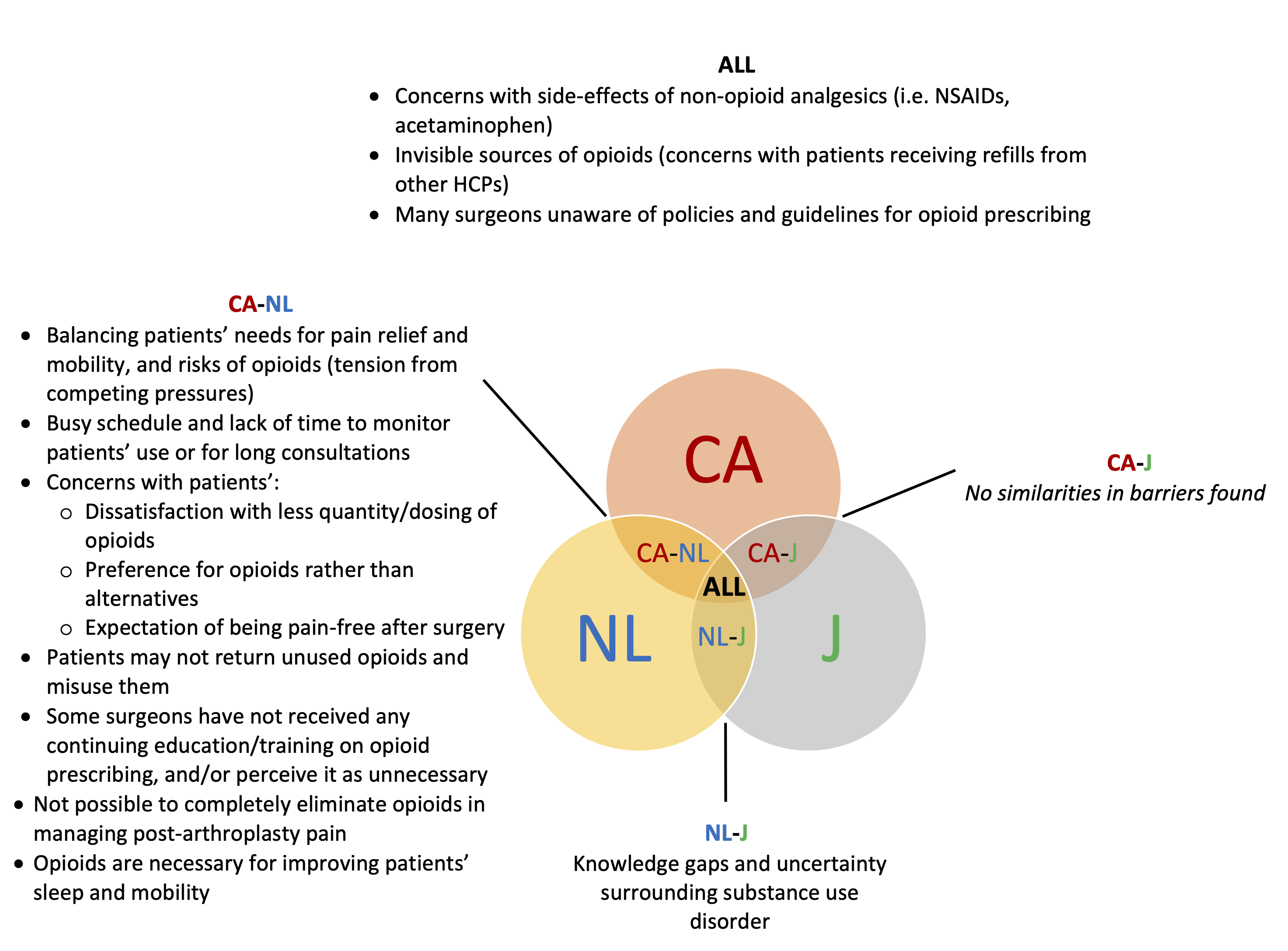

Supplement: S2 Fig — (TIFF) [file pone.0331335.s005.tiff]
