# Supplementary material for: Perceptions of barriers and facilitators to opioid reduction after total joint arthroplasty among orthopedic surgeons practicing in Canada, Japan, and the Netherlands: A qualitative description study
Source: PLoS One. 2025 Aug 29;20(8):e0331335. doi: 10.1371/journal.pone.0331335 (PMC12396690; doi:10.1371/journal.pone.0331335)
Supplement: Attachment — Submitted filename: OreosQual_RevisionLetter 23Jan25.docx [file pone.0331335.s008.docx]

January 23^rd^, 2025

**RE: REVISION: Perceptions of barriers and facilitators to opioid reduction after total joint arthroplasty among orthopedic surgeons practicing in Canada, Japan, and the Netherlands: A qualitative description study**

Dear Dr. Chenette,

Please accept our revisions for the manuscript submission entitled, “Perceptions of barriers and facilitators to opioid reduction after total joint arthroplasty among orthopedic surgeons practicing in Canada, Japan, and the Netherlands: A qualitative description study” for consideration for publication in PLOS One.

We would like to thank the reviewers and editors for their time and effort in reviewing our manuscript and providing comments. We appreciate the quality of the feedback and have edited our manuscript to align with your comments as much as possible, providing justifications where required.

**Journal Requirements:**

When submitting your revision, we need you to address these additional requirements.

1. Please ensure that your manuscript meets PLOS ONE's style requirements, including those for file naming. The PLOS ONE style templates can be found at

<https://journals.plos.org/plosone/s/file?id=wjVg/PLOSOne_formatting_sample_main_body.pdf> and

<https://journals.plos.org/plosone/s/file?id=ba62/PLOSOne_formatting_sample_title_authors_affiliations.pdf>

Thank you, we have re-checked the formatting to ensure it aligns with the requirements.

2. Please include a complete copy of PLOS’ questionnaire on inclusivity in global research in your revised manuscript. Our policy for research in this area aims to improve transparency in the reporting of research performed outside of researchers’ own country or community. The policy applies to researchers who have travelled to a different country to conduct research, research with Indigenous populations or their lands, and research on cultural artefacts. The questionnaire can also be requested at the journal’s discretion for any other submissions, even if these conditions are not met.  Please find more information on the policy and a link to download a blank copy of the questionnaire here: <https://journals.plos.org/plosone/s/best-practices-in-research-reporting>. Please upload a completed version of your questionnaire as Supporting Information when you resubmit your manuscript.

Thank you for this information. We have completed this questionnaire and have submitted it as a supplementary information file (S4 File). We referred to this file on lines 174-176.

a. In the Methods ethics statement, you specified that verbal consent was obtained. Please provide additional details regarding how this consent was documented and witnessed, and state whether this was approved by the IRB.

Thank you. We have added further details in the Methods section, lines 131-137, “Prior to data collection, participants were provided with a research ethics board (REB)-approved information letter that described the study in accessible language and provided sufficient information for participants to make an informed decision about their participation. They were given ample time to review and ask questions. Thereupon, verbal informed consent was obtained during the interview and documented on a REB-approved verbal informed consent log for audio and/or video recording and the use of quotes.”

For further clarity, all participants included in this study reviewed a REB-approved information letter before data collection that described this study in accessible language and provided sufficient information for participants to make an informed decision about their participation. The information letter described the study purpose, detailed background information, the nature of participation, and associated risks and benefits in lay language. Participants were also provided with the contact information of the study investigators, the principal investigator(s), and individual(s) outside the research team who can be contacted regarding questions or possible ethical issues. Then, verbal informed consent was obtained during the interview and documented on a verbal informed consent log, approved by the local IRB.

3. Thank you for stating the following in the Competing Interests section: [I have read the journal's policy and the authors of this manuscript have the following competing interests:

One of the authors (MP) has received funding from the Mitacs Globalink Research Award

One of the authors (VK) is a consultant for Stryker Canada and Zimmer Biomet Canada, and has a spouse who works for Stryker Canada.

One of the authors (YK) received a grant from Pioneers in Healthcare, received honoraria from Agnovos, received support for meeting attendance from RGS, and payment from NOV for participation in a Data Safety Monitoring Board or Advisory Board.

One of the authors (KM) received a grant from CIHR (BioTalent), is a participant in a Data Safety Monitoring Board or Advisory Board at the University of Calgary, and has a leadership or fiduciary role at the Clinical Orthopaedics and Related Research and Canadian Orthopaedic Association.

All other authors (PP, SS, AA, NS, EL, HS, JWB) certify that there are no funding or commercial associations (consultancies, stock ownership, equity interest, patent/licensing arrangements, etc.) that might pose a conflict of interest in connection with the submitted article related to the author or any immediate family members.].

a. Please confirm that this does not alter your adherence to all PLOS ONE policies on sharing data and materials, by including the following statement: "This does not alter our adherence to PLOS ONE policies on sharing data and materials.” (as detailed online in our guide for authors <http://journals.plos.org/plosone/s/competing-interests>). If there are restrictions on sharing of data and/or materials, please state these. Please note that we cannot proceed with consideration of your article until this information has been declared. Please include your updated Competing Interests statement in your cover letter; we will change the online submission form on your behalf.

Thank you for letting us know. We have included the Competing Interests statement along with the aforementioned statement in our revised cover letter.

“Please note the following competing interests: One of the authors (MP) has received funding from the Mitacs Globalink Research Award. One of the authors (VK) is a consultant for Stryker Canada and Zimmer Biomet Canada, and has a spouse who works for Stryker Canada. One of the authors (YK) received a grant from Pioneers in Healthcare, received honoraria from Agnovos, received support for meeting attendance from RGS, and payment from NOV for participation in a Data Safety Monitoring Board or Advisory Board. One of the authors (KM) received a grant from CIHR (BioTalent), is a participant in a Data Safety Monitoring Board or Advisory Board at the University of Calgary, and has a leadership or fiduciary role at the Clinical Orthopaedics and Related Research and Canadian Orthopaedic Association. All other authors (PP, SS, AA, NS, EL, HS, JWB) certify that there are no funding or commercial associations (consultancies, stock ownership, equity interest, patent/licensing arrangements, etc.) that might pose a conflict of interest in connection with the submitted article related to the author or any immediate family members. This does not alter our adherence to PLOS ONE policies on sharing data and materials.”

4. In this instance it seems there may be acceptable restrictions in place that prevent the public sharing of your minimal data. However, in line with our goal of ensuring long-term data availability to all interested researchers, PLOS’ Data Policy states that authors cannot be the sole named individuals responsible for ensuring data access (<http://journals.plos.org/plosone/s/data-availability#loc-acceptable-data-sharing-methods>). Data requests to a non-author institutional point of contact, such as a data access or ethics committee, helps guarantee long term stability and availability of data. Providing interested researchers with a durable point of contact ensures data will be accessible even if an author changes email addresses, institutions, or becomes unavailable to answer requests. Before we proceed with your manuscript, please also provide non-author contact information (phone/email/hyperlink) for a data access committee, ethics committee, or other institutional body to which data requests may be sent. If no institutional body is available to respond to requests for your minimal data, please consider if there any institutional representatives who did not collaborate in the study, and are not listed as authors on the manuscript, who would be able to hold the data and respond to external requests for data access? If so, please provide their contact information (i.e., email address). Please also provide details on how you will ensure persistent or long-term data storage and availability.

Thank you. We have submitted all data to MacSphere, McMaster University's open-access institutional repository. Upon approval, this will ensure long-term storage and accessibility of the data. For any data requests, please contact the McMaster University Office of Scholarly Communication at [scom@mcmaster.ca](mailto:scom@mcmaster.ca).

5. Please upload a copy of Figures 1 and 2, to which you refer in your text on page 10. If the figure is no longer to be included as part of the submission please remove all reference to it within the text.

Thank you for letting us know. We have uploaded S3 Fig 1 and S3 Fig 2 (included in S3 File) separately as well.

6. Please review your reference list to ensure that it is complete and correct. If you have cited papers that have been retracted, please include the rationale for doing so in the manuscript text, or remove these references and replace them with relevant current references. Any changes to the reference list should be mentioned in the rebuttal letter that accompanies your revised manuscript. If you need to cite a retracted article, indicate the article’s retracted status in the References list and also include a citation and full reference for the retraction notice.

We have reviewed the reference list and ensure that it is complete and correct.

**Reviewers' Comments:**

1. Is the manuscript technically sound, and do the data support the conclusions?

   The manuscript must describe a technically sound piece of scientific research with data that supports the conclusions. Experiments must have been conducted rigorously, with appropriate controls, replication, and sample sizes. The conclusions must be drawn appropriately based on the data presented.
   a. Reviewer #1: Yes
   b. Reviewer #2: Yes

Thank you.

2. Has the statistical analysis been performed appropriately and rigorously?
   a. Reviewer #1: Yes
   b. Reviewer #2: I Don't Know

Thank you. Due to the qualitative nature of this study, data analysis involved conventional (inductive) content analysis. We summarized demographic data using ranges and proportions.

3. Have the authors made all data underlying the findings in their manuscript fully available? The [PLOS Data policy](http://track.editorialmanager.com/CL0/http:%2F%2Fwww.plosone.org%2Fstatic%2Fpolicies.action%23sharing/1/010f0193cd3eeec0-1e0ca214-9006-4245-8622-d95089c96460-000000/0SHgAm4rjtvLKs7Ys_iUeSdFJzDW8dA8NFXiDLuFwbI=189) requires authors to make all data underlying the findings described in their manuscript fully available without restriction, with rare exception (please refer to the Data Availability Statement in the manuscript PDF file). The data should be provided as part of the manuscript or its supporting information, or deposited to a public repository. For example, in addition to summary statistics, the data points behind means, medians and variance measures should be available. If there are restrictions on publicly sharing data—e.g. participant privacy or use of data from a third party—those must be specified.
   a. Reviewer #1: Yes
   b. Reviewer #2: No

To protect the privacy or confidentiality of human research participants, not all data can be made available publicly (i.e. direct quotations of participants that may reveal their identity). Interested researchers can contact the corresponding author or McMaster University Office of Scholarly Communication at [scom@mcmaster.ca](mailto:scom@mcmaster.ca) to access the minimal data that we have. They can also access this data at <https://macsphere.mcmaster.ca>.

4. Is the manuscript presented in an intelligible fashion and written in standard English? PLOS ONE does not copyedit accepted manuscripts, so the language in submitted articles must be clear, correct, and unambiguous. Any typographical or grammatical errors should be corrected at revision, so please note any specific errors here.
   a. Reviewer #1: Yes
   b. Reviewer #2: Yes

Thank you.

**Review Comments to the Author:**

Please use the space provided to explain your answers to the questions above. You may also include additional comments for the author, including concerns about dual publication, research ethics, or publication ethics. (Please upload your review as an attachment if it exceeds 20,000 characters)

5. **Reviewer #1:** This article addresses a critical and timely issue of reducing opioid use following joint arthroplasty, providing valuable insights through a cross-cultural comparison of prescribing practices in different countries.

Thank you for your thorough feedback and taking the time to review our manuscript.

The article could be improved with the following suggestions:

a. The results compare countries but lack sufficient emphasis on the unique cultural and systemic factors influencing opioid prescribing behaviors. The suggestion of providing more detailed comparisons between countries focuses on how cultural and systemic differences shape prescribing practices. Including a table or figure summarizing these differences would improve clarity.

The results summarize the perceptions of orthopaedic surgeons that we identified through the interview sessions. Considering the qualitative description methodology used in the study, we provided an analytical and data-driven perspective, by using conventional (inductive) content analysis, rather than interpreting the findings. We highlighted differences and similarities in the perceptions of orthopaedic surgeons identified during interviews from each of the 3 countries in S3 Tables 2 to 7 and S3 Fig 1 and S3 Fig 2.

b. The discussion could benefit from stronger connections to existing literature, particularly around the role of national policies and patient education in opioid prescribing.

Thank you for your feedback. We have discussed the role of national policies in opioid prescribing in Japan (Lines 374-387). We have also added the following to the discussion:

- “Notably, a retrospective cohort study, evaluating postoperative opioid prescription quantities, variability, and 30-day refill rates before and after implementation of orthopedic procedure-specific guidelines reported decreased opioid prescription amounts and variability” (Lines 406-409).
- “Moreover, this underscores the continued importance of educating patients on the risks associated with opioids, expectations of pain management, exploring alternative treatment options, and providing guidance on safe handling and proper disposal methods. A systematic review of 11 studies, evaluating the effectiveness of pre-operative patient education on post-operative opioid use and pain management in the orthopaedic setting, found that education related to opioid use and pain can be effective in reducing opioid prescription requests and filling[36]. Similarly, a cluster-randomized controlled trial including 539 patients reported that patient education on safe opioid disposal approximately triples opioid disposal rates compared with no education [33].” (Lines 349-358).
- “Similarly, many European countries enforce regulatory restrictions on the prescribing of opioids including dose limits, duplicate or triplicate prescriptions using special forms, and strict monitoring by pharmacies [39]” (Lines 387-389).
  c. While the study identifies barriers, it lacks concrete recommendations for overcoming them. Suggestions for specific, actionable strategies tailored to each country, such as surgeon training programs, standardized protocols, or policy initiatives.

The scope of this qualitative study is limited as it serves as a preliminary exploration with a small sample size, and our findings may not generalizable. We have acknowledged this in the limitations section of the study, “As qualitative studies seek to understand a specific phenomenon, generalizability is not an expected attribute as it is for quantitative studies.” (Lines 426-428).

We have now acknowledged this issue in our Conclusion as well, “Rigorous quantitative studies, with larger sample sizes, are needed to validate these qualitative findings and assess their generalizability across broader populations. Additionally, further qualitative and quantitative studies, exploring patient perspectives on opioid use, are needed for achieving a more comprehensive understanding of the issue.” (Lines 439-443).

If the findings of this qualitative study were found to be generalizable, potential strategies could include implementing surgeon training programs, developing standardized opioid prescribing protocols, introducing policy initiatives tailored to each country’s healthcare context, and enhancing patient education.

We have also added the following to our discussion section, which outlines existing guidelines and positions to guide opioid prescribing among surgeons. “Most surgeons were aware of institutional regulations and restrictions on opioid prescribing, but many surgeons in Canada and the Netherlands lacked knowledge of existing guidelines and policies. The Canadian Orthopedic Association’s position statement on opioid prescribing highlights the use of the 2017 Canadian Guideline for Opioids for Chronic Non-Cancer Pain which offers direction for physicians in Canada regarding the prescription of opioids and advocates for a more conservative approach[38, 39]. This guideline is complementary to several province-specific resources and practice standards such as the Safe Prescribing of Opioids and Sedatives practice standard by the College of Physicians and Surgeons of British Columbia and the Public Health Ontario Interactive Opioid Tool[40]. Moreover, the CDC Clinical Practice Guideline for Prescribing Opioids for Pain — United States, 2022, provides recommendations for clinicians prescribing opioids to adult outpatients[41]. Recently, clinical practice recommendations were developed for prescribing opioids to patients with chronic noncancer pain in Europe[42]. However, there is a need for surgery-specific guidelines on opioid prescribing to promote evidence-based practices.” (Lines 391-409).

d. Limitations Section: The authors acknowledge some limitations (e.g., gender imbalance), but others are understated, such as potential biases arising from interviewing surgeons who may already hold strong views on opioid prescribing.

Thank you. This is addressed in the first line of our Limitations section, “Our study involved surgeons who volunteered to participate and, thus, may have had a heightened interest or stronger perceptions regarding opioid use and prescribing.” (Lines 420-421).

e. Further suggestion is to address the strengths of this study.

Thank you for your suggestion. We have added the strengths of our study, “Our study provides a cross-cultural understanding of opioid prescribing practices by exploring the perspectives of orthopedic surgeons across three countries that represent high (Canada), moderate (the Netherlands), and low (Japan) opioid prescribing practices. The qualitative approach allows for an in-depth exploration of nuanced barriers and facilitators that may not be captured through quantitative methods. Additionally, we implemented triangulation and member checking to enhance the trustworthiness of our findings” (Lines 412-417).

Additional Recommendations

f. Incorporate Practical Insights:
   i. Highlight how the findings can directly inform policies or training programs for surgeons.

This qualitative study serves as a preliminary exploration with a small sample size, aimed at gaining an in-depth understanding of the phenomenon. Given the study's scope and methodological approach, the findings may not be generalizable (Lines 426-428). To inform policies and training programs for surgeons, rigorous, large quantitative studies will be necessary to validate these findings, assess their generalizability, and provide a robust evidence base for informing policies or training programs (Lines 439-443).

g. Ethical Considerations:
   Elaborate on measures to ensure confidentiality and address potential power dynamics during interviews, which could influence participants' responses.

We are unaware of a power imbalance that could have influenced the participants’ responses. The interviews were conducted by graduate and undergraduate students who had no authoritative position or influence over the participants' careers. Measures were also taken to ensure confidentiality, allowing participants to share their perspectives openly and without concern for professional repercussions. We have added this in the Methods section as well, “Potential power imbalances were minimized as interviews were conducted by graduate and undergraduate students with no authority or influence over participants' careers.” (Lines 169-170).

h. Future Research Directions:
   Discuss the need for follow-up quantitative studies to validate the qualitative findings and explore patient perspectives to provide a more comprehensive understanding of the issue.

Thank you for your feedback. We have added that, “Rigorous quantitative studies, with larger sample sizes, are needed to validate these qualitative findings and assess their generalizability across broader populations. Additionally, further qualitative and quantitative studies, exploring patient perspectives on opioid use, are needed for achieving a more comprehensive understanding of the issue” in the Conclusion (Lines 439-443).

6. **Reviewer #2:** I would like to thank all the authors for their efforts in conducting this quality study. Nearly all the key issues and limitations associated with this research have been thoughtfully acknowledged and addressed within the manuscript itself.

Thank you for taking the time to review our manuscript.

Thank you.
