# Supplementary material for: Perceptions of barriers and facilitators to opioid reduction after total joint arthroplasty among orthopedic surgeons practicing in Canada, Japan, and the Netherlands: A qualitative description study
Source: PLoS One. 2025 Aug 29;20(8):e0331335. doi: 10.1371/journal.pone.0331335 (PMC12396690; doi:10.1371/journal.pone.0331335)
Supplement: Attachment — Submitted filename: OreosQual_RevisionLetter 18Jun25.docx [file pone.0331335.s009.docx]

June 18^th^, 2025

**RE: REVISION 2: Perceptions of barriers and facilitators to opioid reduction after total joint arthroplasty among orthopedic surgeons practicing in Canada, Japan, and the Netherlands: A qualitative description study**

Dear Dr. Chenette,

Please accept our revisions for the manuscript submission entitled, “Perceptions of barriers and facilitators to opioid reduction after total joint arthroplasty among orthopedic surgeons practicing in Canada, Japan, and the Netherlands: A qualitative description study” for consideration for publication in PLOS One.

We would like to thank the reviewers and editors for their time and effort in reviewing our manuscript and providing comments. We appreciate the quality of the feedback and have edited our manuscript to align with your comments as much as possible, providing justifications where required.

**Journal Requirements:**
Please review your reference list to ensure that it is complete and correct. If you have cited papers that have been retracted, please include the rationale for doing so in the manuscript text or remove these references and replace them with relevant current references. Any changes to the reference list should be mentioned in the rebuttal letter that accompanies your revised manuscript. If you need to cite a retracted article, indicate the article’s retracted status in the References list and also include a citation and full reference for the retraction notice.
We have checked the reference list and ensure that it is up-to-date and complete.

**Reviewers' comments:**

1. If the authors have adequately addressed your comments raised in a previous round of review and you feel that this manuscript is now acceptable for publication, you may indicate that here to bypass the “Comments to the Author” section, enter your conflict-of-interest statement in the “Confidential to Editor” section, and submit your "Accept" recommendation.

Reviewer #1: All comments have been addressed

Thank you.

2. Is the manuscript technically sound, and do the data support the conclusions?
   The manuscript must describe a technically sound piece of scientific research with data that supports the conclusions. Experiments must have been conducted rigorously, with appropriate controls, replication, and sample sizes. The conclusions must be drawn appropriately based on the data presented.

Reviewer #1: Yes

Thank you.

3. Has the statistical analysis been performed appropriately and rigorously?

Reviewer #1: Yes

Thank you.

4. Have the authors made all data underlying the findings in their manuscript fully available?

The PLOS Data policy requires authors to make all data underlying the findings described in their manuscript fully available without restriction, with rare exception (please refer to the Data Availability Statement in the manuscript PDF file). The data should be provided as part of the manuscript or its supporting information, or deposited to a public repository. For example, in addition to summary statistics, the data points behind means, medians and variance measures should be available. If there are restrictions on publicly sharing data—e.g. participant privacy or use of data from a third party—those must be specified.

Reviewer #1: Yes

Thank you.

5. Is the manuscript presented in an intelligible fashion and written in standard English?
   PLOS ONE does not copyedit accepted manuscripts, so the language in submitted articles must be clear, correct, and unambiguous. Any typographical or grammatical errors should be corrected at revision, so please note any specific errors here.

Reviewer #1: Yes

Thank you.

6. Review Comments to the Author
   Please use the space provided to explain your answers to the questions above. You may also include additional comments for the author, including concerns about dual publication, research ethics, or publication ethics. (Please upload your review as an attachment if it exceeds 20,000 characters)

Reviewer #1:

Dear Authors,

Thank you for thoroughly addressing the previous review comments. Your revisions have significantly improved the manuscript, particularly in the areas of cultural context, policy implications, and study limitations. I appreciate your efforts in providing additional data and strengthening the discussion section.
Thank you for your feedback.

Final Recommendations:
Practical Application:

While you have acknowledged the preliminary nature of this study, including a brief section on how these findings could inform clinical guidelines or surgeon training would enhance the manuscript’s real-world impact.

Thank you for your feedback. We have added the following section in the Discussion:

“**Cross-national insights can be integrated into the development of surgery-specific opioid prescribing guidelines. The variability observed across Canada, the Netherlands, and Japan highlights the need for evidence-based protocols that account for both cultural contexts and patient preferences. Our findings suggest that surgeon training programs can integrate updated guidance on multimodal pain management strategies, safe opioid prescribing, and the importance of educating patients on proper storage and disposal of unused opioid medications. Emphasizing existing national and international guidelines within training can further support consistent, informed decision-making. Addressing the identified barriers will require a multifaceted approach, including system-level changes and targeted educational interventions.** Future research can evaluate the effectiveness of such strategies in supporting surgeons to adopt patient-centered, evidence-based practices that minimize opioid use while ensuring adequate pain control. Multi-center, quantitative studies are also needed to improve generalizability and explore cultural influences on prescribing behavior. Additionally, qualitative and quantitative studies exploring patient perspectives may offer a more holistic understanding of pain management preferences and inform more tailored interventions.” (Lines 404-418).

Future Research Directions:

Your inclusion of future research needs is appreciated. However, a more explicit mention of potential multi-center studies or patient perspectives could further guide subsequent investigations in this field.
Thank you for your feedback. We have added the following section in the Discussion:

“Cross-national insights can be integrated into the development of surgery-specific opioid prescribing guidelines. The variability observed across Canada, the Netherlands, and Japan highlights the need for evidence-based protocols that account for both cultural contexts and patient preferences. Our findings suggest that surgeon training programs can integrate updated guidance on multimodal pain management strategies, safe opioid prescribing, and the importance of educating patients on proper storage and disposal of unused opioid medications. Emphasizing existing national and international guidelines within training can further support consistent, informed decision-making. Addressing the identified barriers will require a multifaceted approach, including system-level changes and targeted educational interventions. **Future research can evaluate the effectiveness of such strategies in supporting surgeons to adopt patient-centered, evidence-based practices that minimize opioid use while ensuring adequate pain control. Multi-center, quantitative studies are also needed to improve generalizability and explore cultural influences on prescribing behavior. Additionally, qualitative and quantitative studies exploring patient perspectives may offer a more holistic understanding of pain management preferences and inform more tailored interventions**.” (Lines 404-418).

Overall, the manuscript now meets the scientific and editorial standards for publication, and I recommend acceptance.
Thank you for your dedication to advancing this important area of research.

Thank you for your feedback.

Thank you.
